# Custodiol *versus* Blood Cardioplegia: Comparison of Myocardial Immunohistochemical Analysis and Clinical Outcomes

**DOI:** 10.21470/1678-9741-2020-0662

**Published:** 2022

**Authors:** Onur Sen, Unal Aydin, Ersin Kadirogullari, Salih Güler, Süheyla Gonca, Seyhun Solakoğlu, Mehmet Karaçalılar, Barış Timur, Burak Onan

**Affiliations:** 1 Department of Cardiovascular Surgery, Istanbul Mehmet Akif Ersoy Thoracic and Cardiovascular Surgery Training and Research Hospital, Istanbul, Turkey; 2 Department of Cardiovascular Surgery, GATA, Ankara, Turkey; 3 Department of Histology and Embryology, Kocaeli University School of Medicine, Kocaeli, Turkey; 4 Department of Histology and Embryology, Istanbul University School of Medicine, Istanbul, Turkey

**Keywords:** Custodiol-N solutions, Cardioplegic Solutions, Oxidative Stress, Cardiac Surgical Procedures, Heart Arrest, Induced, Immunohistochemistry

## Abstract

**Introduction:**

Custodiol (histidine-tryptophan-ketoglutarate) and repetitive blood cardioplegia are the solutions for myocardial protection and cardiac arrest. In this study, we aimed to compare immunohistochemical analysis, clinical outcomes, and cardiac enzyme values of Custodiol and blood cardioplegia groups.

**Methods:**

This was a randomized prospective study consisting of 2 groups and 20 patients, 10 patients for each group, who underwent mitral and mitral/tricuspid valve surgery. Group 1 was formed for Custodiol cardioplegia and group 2 for blood cardioplegia. Perioperative and postoperative cardiac events were recorded, cardiac enzymes were analyzed with intervals, and myocardial samples were taken for immunohistochemical analysis. Recorded data were statistically evaluated.

**Results:**

There was no significant difference for the Custodiol and blood cardioplegia groups in perioperative and postoperative cardiac performance and adverse events. Cardiac enzyme analysis showed no significant difference between groups. However, two parameters (eNOS, Bcl-2) were in favor of the Custodiol group in immunohistochemical studies. Custodiol performed better in cellular oxidative stress resistance and cellular viability.

**Conclusion:**

Clinical outcomes and cardiac enzyme analysis results were similar regarding myocardial protection. However, Custodiol performed better in the immunohistochemical analysis.

**Table:** 

Abbreviations, Acronyms & Symbols	
AEC	= Aminoethyl carbazole
ATP	= Adenosine triphosphate
Bcl-2	= B-cell lymphoma 2
CPB	= Cardiopulmonary bypass
ECG	= Electrocardiography
eNOS	= Endothelial nitric oxide synthase
ICU	= Intensive care unit
iNOS	= Inducible nitric oxide synthase
LV	= Left ventricular
NO	= Nitric oxide
SD	= Standard deviation
SPSS	= Statistical Package for the Social Sciences
VEGF	= Vascular endothelial growth factor
VF	= Ventricular fibrillation

## INTRODUCTION

Myocardial protection is one of the most important issues in cardiac surgery, and cardioplegic solutions improve the tolerance to ischemia and protect against the adverse effects of reperfusion. Custodiol (histidine-tryptophan-ketoglutarate) is an intracellular crystalloid cardioplegic solution that provides diastolic cardiac arrest via hyperpolarization of the myocyte plasma membrane. Consequently, Custodiol improves high energy production, stabilizes cell membranes, and maintains osmotic regulation of the cell membrane. In recent years, Custodiol has been used as an organ preservation solution for cardiac transplantation, as well as other organs^[1]^. Custodiol was developed by Bretschneider in the 1970s and has been used as a cardioprotective solution for cardiac surgery and transplantation^[2,3]^. The components of Custodiol act as a multifunctional protection mechanism; histidine buffers the acidosis accumulated by anaerobic metabolism, tryptophan stabilizes the cell membrane, ketoglutarate improves adenosine triphosphate (ATP) production during reperfusion, and mannitol decreases cellular edema^[2]^.

Blood cardioplegia is a routine and conventional solution that has been proposed as a safe and reliable technique for myocardial protection. Its high potassium content causes diastolic arrest via myocyte membrane depolarization. Blood, as a medium for cardioplegia delivery, has a greater oxygen-carrying capacity and is less associated with hemodilution^[4]^. The composition of Custodiol and blood cardioplegia is listed in Table 1. Currently, there is no consensus as to the optimal cardioplegic solution delivery. Therefore, we aimed to compare the histopathological and clinical outcomes of Custodiol *versus* blood cardioplegia solutions.

**Table 1:** Composition of Custodiol *versus* blood cardioplegia.

	Custodiol	Blood cardioplegia
Na^+^	15 mmol/L	140 mmol/L
K^+^	9 mmol/L	20-10 mmol/L
Mg^+^2^^	4 mmol/L	--
Ca^+^2^^	0.015 mmol/L	--
Histidine	198 mmol/L	--
Tryptophan	2 mmol/L	--
Ketoglutarate	1 mmol/L	--
Mannitol	30 mmol/L	--
Glucose	--	6 mmol/L
pH	7.02-7.20	7.20-7.40

## METHODS

This study was designed as a prospective study and performed on 20 patients who underwent mitral valve surgery. Patients were included in this study upon receipt of informed written consent. The study protocol was approved by the local ethics committee (Mehmet Akif Ersoy Clinical Research Ethics Committee). Demographic data and risk factors are listed in Table 2. Patients were divided into two groups according to the cardioplegia solution used. Cardioplegia solutions were designated as Custodiol in group 1 (n=10) and blood cardioplegia in group 2 (n=10). Patients were randomly included in the groups preoperatively. Group 1 received a single dose of antegrade cold (4°C) Custodiol solution and group 2 received repetitive antegrade isothermic (34°C) blood cardioplegic solution. No retrograde cardioplegia and additional topical cooling were used in any patient.

**Table 2:** Demographic data and risk factors.

	Custodiol	Blood cardioplegia	*P*
Age	48.9±10.05	49.9±19.2	0.886
Sex (M/F)	4/6	1/9	0.121
Hypertension	2	2	0.999
Diabetes mellitus	2	2	0.999
Hypercholesterolemia	2	1	0.531
Chronic pulmonary disease	3	2	0.606
Creatinine >1.2	1	1	0.999
Atrial fibrillation	0	2	0.136
Coronary artery disease	1	0	0.305
Left ventricular dysfunction	2	1	0.531
Cerebrovascular disease	0	1	0.305
Smoking	5	3	0.361

Conventional general anesthesia was used in all patients. Mitral valve surgery was performed via a left atriotomy with a median sternotomy. All surgeries were performed using cardiopulmonary bypass (CPB) with the use of a roller pump, ascending aortic cannulation, double venous cannulation, and moderate systemic hypothermia (32-34°C). Anticoagulation was provided through systemic heparinization and CPB was initiated in the process of activated clotting time over 400 seconds. Patients who required arrhythmia surgery were excluded from the study.

Isothermic blood cardioplegia was delivered antegradely through a cannula placed in the ascending aorta every 20 minutes with a pressure of 200-250 mmHg at the time of aortic cross-clamping. A total of 900 mL of blood cardioplegia was administered to the patient initially. Thereafter, 500 mL of blood cardioplegia was given antegradely every 20 minutes^[5]^. During the delivery, aortic valve competence was checked through digital palpation of the pressure in the ascending aorta and volume overload at the left ventricle. Custodiol was administered antegradely through the ascending aorta at an initial perfusion pressure of 80-100 mmHg at the time of 6-8 minutes as described by the manufacturer. Each patient received 20-25 mL of Custodiol per kg of body weight, in accordance with previous studies^[6]^. Atriotomies were performed after cardiac arrest was sustained. Therefore, Custodiol coming from the coronary sinus went directly to the CPB reservoir. Ultrafiltration was used for patients receiving Custodiol. Twenty percent of the patients had mild hyponatremia after using Custodiol. Postoperative 0, 1^st^, 3^rd^, and 6^th^ hours arterial blood gas analyses were routinely performed, and electrolyte values were closely followed. All electrolyte imbalances were normalized within the first 24 hours.

Continuous telemetry monitoring was used for every patient from the beginning of the surgery until discharge. Intra- and postoperative adverse events were recorded and listed in Tables 3 and 4.

**Table 3:** Operative data.

	Custodiol	Blood cardioplegia	*P*
**Perioperative**			
Mitral valve surgery	7	8	0.606
Concomitant mitral and tricuspid valve surgery	3	2	0.605
CPB time	101.9±12.05 (85-140)	94.0±18.59 (87-132)	0.273
Cross-clamp time	83.30±11.08 (72-112)	76.90±15.56 (70-121)	0.304
**Postoperative**			
Myocardial infarction	0	0	0.999
Atrial fibrillation	2	2	0.999
Ventricular fibrillation	0	0	0.999
New-onset LV dysfunction	0	0	0.999
New-onset nephropathy	0	0	0.999
Prolonged ventilation	0	0	0.999
High-dose inotropic support	0	1	0.305
IABP insertion	0	0	0.999
ICU stay (hours)	38.1±30.2	26.8±7.8	0.268
Hospital stay (days)	7.2±2.93	7.30±0.9	0.920
Mortality	0	0	0.999

CPB=cardiopulmonary bypass; IABP=intra-aortic balloon pump; ICU=intensive care unit; LV=left ventricular

**Table 4:** Comparison of cardiac enzymes in the postoperative 6^th^, 12^th^ and 24^th^ hours.

	Custodiol	Blood cardioplegia	*P*
**CK-MB**			
6^th^ hour	39.43±9.3	45,43±12.3	0.345
12^th^ hour	34.62±7.38	43.24±22.03	0.256
24^th^ hour	32.43±9.34	37.6±12.34	0.451
**Troponin I**			
6^th^ hour	0.487±0.189	0.554±0.291	0.405
12^th^ hour	0.487±0.177	0.908±1.228	0.298
24^th^ hour	0.385±0.158	1.053±1.906	0.284

Troponin I (Tn I) and creatine kinase isoenzyme MB (CK-MB) were defined as equivalence in surgical outcomes between Custodiol and blood cardioplegia. Blood samples for determination of Tn I and CK-MB were obtained 6, 12, and 24 hours following the release of the cross-clamp (Table 4). These molecules were studied in the central laboratory (Cobas-c501 Roche, USA, 0-0.014 ng/mL) of the hospital.

### Immunohistochemical Study Protocol

After completing the mitral valve procedure, a left atrial tissue sample was taken just before cross-clamp removal and the sample was stored in paraformaldehyde at 4°C. Immunohistochemistry was performed (formalin/PFA-fixed paraffin-embedded sections) using the avidin-biotin-peroxidase method (Zymed, San Francisco, CA). Sections were incubated in iNOS (ready-to-use, prediluted. Thermo Scientific, UK) Bcl-2 (ready-to-use, prediluted. Novocastra Laboratories, UK), VEGF (NeoMarkers, 1:50), annexin (Zeta, 1:100) and eNOS (Anti-eNOS antibody, 200 µl, Abcam) for 24h at 4°C in a humidified chamber. Aminoethyl carbazole (AEC) is the chromogen of choice when performing immunoperoxidase staining for 5 minutes at room temperature. After counterstaining with Mayer's hematoxylin, immunoreactivity was examined using a light microscope (Leica DM6000 B; Leica Microsystems Inc.; Buffalo Grove, Ill).

In each group, the intensity of the positive immune stained cells in each section was assessed by visual observation. Immunoreactivity was then graded according to a 4-degree semiquantitative scale: minimal immunostaining (+), mild immunostaining (++), moderate immunostaining (+++), or severe immunostaining (++++).

### Statistical Analysis

Statistical analysis was performed using the statistical software SPSS version 23.0 for Windows (SPSS Inc., Chicago, IL, USA). Data distribution was studied using the Kolmogorov-Smirnov test. Normally distributed continuous variables are expressed as mean±standard deviation (SD), and categorical variables are expressed as numbers and percentages. Continuous variables were compared using either the independent samples t-test or the Mann-Whitney U test. The chi-square test was used to compare categorical variables. The *P*-value was two-tailed with a significance level of 0.05.

## RESULTS

Demographic data and risk factors are evaluated in Table 2 for blood cardioplegia and Custodiol groups. There was no significant difference between groups. Mitral valve surgery (Custodiol group, n=7, blood cardioplegia group, n=8) and mitral valve and concomitant tricuspid valve surgery (Custodiol group, n=3, blood cardioplegia group, n=2) procedures were performed. The mean CPB (101.9±12.05 min *vs.* 94.0±18.59; *P*=0.273 min) and cross-clamp times (83.30±11.08 min *vs.* 76.90±15.56; *P*=0.304 min) were analyzed and the groups were considered similar. After cross-clamp removal, bradycardia requiring temporary pacing was observed in 2 patients from the Custodiol group and 3 from the blood cardioplegia group. A normal rhythm was reestablished on the 2^nd^ postoperative day and pacing leads were extracted before discharge.

During the intensive care unit (ICU) follow-up, no acute myocardial infarction, ventricular fibrillation, new-onset left ventricular dysfunction, new-onset nephropathy, and prolonged ventilation occurred in any patient in either group. Only one patient from the blood cardioplegia group needed high-dose inotropic support for 3 days. Electrocardiography (ECG) studies were performed in all patients postoperatively and no ECG changes occurred in six patients from the Custodiol group and five from the blood cardioplegia group. At rest, atrial fibrillation was diagnosed in two patients from each group, and nonspecific changes were detected in two patients from the Custodiol group and three from the blood cardioplegia group.

**Fig. 1:**
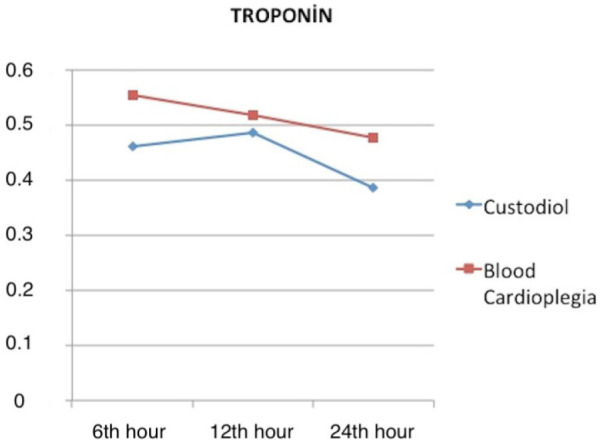
Mean troponin I values for both Custodiol and blood cardioplegia groups in 6^th^, 12^th^ and 24^th^ hours after the cross-clamp was removed.

**Fig. 2:**
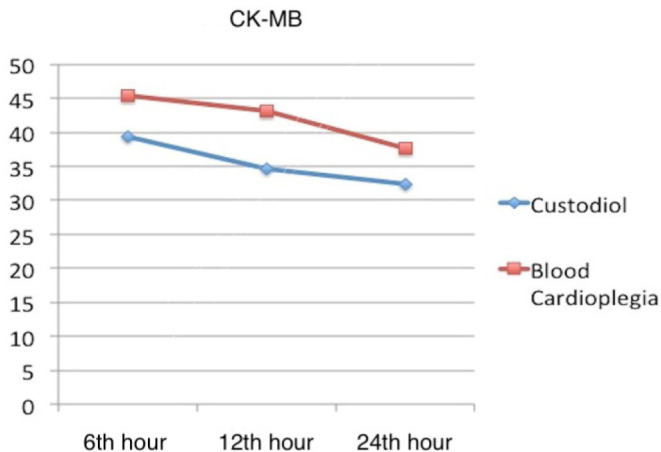
Mean CK-MB values for both Custodiol and blood cardioplegia groups in 6^th^, 12^th^ and 24^th^ hours after the cross-clamp was removed.was removed.

The Tn I and CK-MB plasma levels obtained at the intervals are evaluated in Figures 1 and 2. Similar results were obtained in both groups; no superiority of any solution could be observed via released enzyme concentrations (*P*>0.05). Perioperatively and during ICU follow-up, there were no clinically and/or statistically discernible differences between the two groups.

**Fig. 3:**
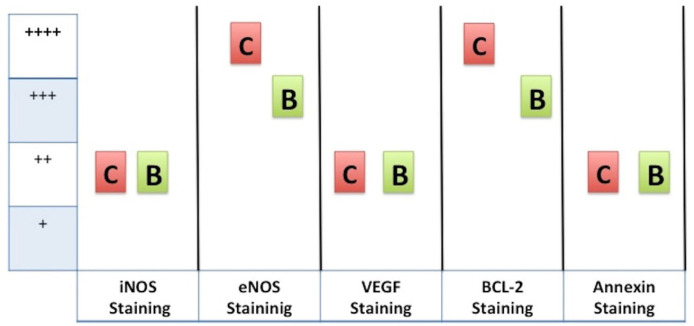
Immunohistochemical markers and median values of staining patterns for Custodiol and blood cardioplegia groups. C: Custodiol group; B: blood cardioplegia group.

**Fig. 4:**
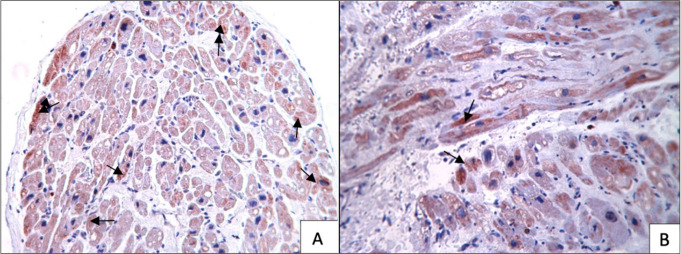
eNOS immunostainings of Custodiol versus Blood cardioplegia samples. (A) Custodiol group. Moderate or intense eNOS immunostaining is seen in this section. (B) Blood cardioplegia group. In this section, mild or moderate eNOS immunostaining is seen. Arrows: eNOS stained cardiomyocytes.

**Fig. 5:**
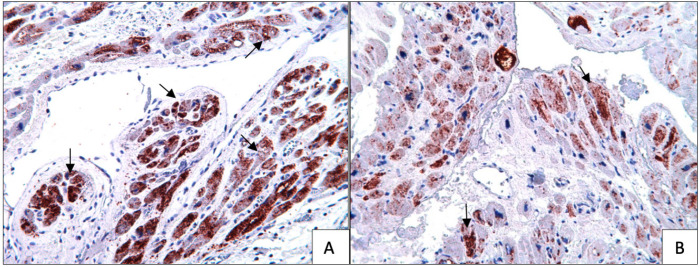
Bcl-2 immunostaining of Custodiol (A) versus blood cardioplegia (B) samples, 20X. (A) Bcl-2 immunostaining is very intense in this section. Custodiol group, 20X. (B) Bcl-2 immunostaining is weak or moderate in this section. Arrows: Bcl-2 stained cardiomyocytes.

Immunohistochemical studies showed moderate eNOS staining (+++), mild iNOS staining (++), mild VEGF staining (++) mild annexin staining (++), and moderate Bcl-2 staining (+++) in the blood cardioplegia group (Figure 3). However, severe eNOS staining (++++), mild iNOS staining (++), mild VEGF staining (++), mild annexin staining (++), and severe Bcl-2 staining (++++) were observed in the Custodiol group (Figure 3). The iNOS, VEGF, and annexin staining were similar in both groups. However, eNOS and Bcl-2 staining were more prominent in the Custodiol group than in the blood cardioplegia group (Figures 4 and 5) (*P*<0.01).

*Minimal immunostaining (+), mild immunostaining (++), moderate immunostaining (+++), or severe immunostaining (++++).*

## DISCUSSION

Blood cardioplegia has been a standard solution for myocardial protection and diastolic arrest. However, Custodiol has gained popularity. These solutions have been compared for clinical outcomes and enzymatic changes; nevertheless, immunohistochemical studies have not been reported in the literature. The focus of this study was to analyze the cellular immunohistochemical results and clinical outcomes of these solutions.

Immunohistochemical studies are performed by analyzing molecules that are active in apoptosis, inflammation, and cell death during the ischemia-reperfusion processes. Inducible nitric oxide synthase (iNOS) is one of the enzymes that generate nitric oxide (NO) from the amino acid l-arginine. Expression of iNOS is induced in response to cytokines and other agents^[7]^. Endothelial nitric oxide synthase (eNOS) is an enzyme that mediates oxidative stress in ischemia/reperfusion injury^[8]^. Also, eNOS is a major isoform that regulates vascular function, and is a powerful vasodilator^[9]^. Vascular endothelial growth factor (VEGF) is the molecule of response to ischemia generated by the cell^[10]^. It is upregulated by hypoxia in cell lines. Bcl-2 is a proto-oncogene molecule that suppresses apoptotic cell death in a variety of *in vitro* systems and cell lines^[11]^. Annexins are a family of phospholipid-binding proteins that prohibit apoptosis and inflammation to mitigate cellular damage^[12]^. Annexin plasma levels increase during ischemia.

In our study, there was no significant difference between groups in demographic data and risk factors. Additionally, the procedures performed were also similar for both groups. As a consequence, clinical outcomes and postoperative adverse events were also similar (Table 3). However, an increase in ventricular fibrillation after cross-clamp removal in patients receiving Custodiol was reported by Braathen et al.^[5]^. This might be a result of heterogeneous myocardial perfusion, oxidative stress, and electrolyte imbalance. The surgeries we performed were not complex and did not involve coronary artery occlusive disease, consequently, ventricular fibrillation (VF) did not occur in our study groups. Myocardial infarction and left ventricular (LV) systolic dysfunction also did not occur in any patient. In our opinion, this was a result of the homogenous distribution of cardioplegia solutions and electrolyte balance. Only one patient required high-dose inotropic support in the blood cardioplegia group, which was temporary. Hachida et al.^[13]^ reported that the promotion of anaerobic glycolysis during Custodiol ischemia resulted in superior prolonged preservation of myocardial contractile functions. The temperatures of cardioplegic solutions were different as 32°C (blood cardioplegia) *versus* 4°C (Custodiol). This is the debate in the study, because Custodiol should be infused at 4°C to prevent myocardial injury and results in different myocardial warming degrees between groups. Different temperatures and solutions were studied by von Oppell et al.^[14]^ and the best solution for myocardial preservation was presented as Custodiol.

Tn I and CK-MB levels and changes in time are good markers of ischemia induced by myocardial degeneration. In this study, myocardial injury biomarkers, Tn I and CK-MB, were analyzed at different time intervals and slight differences occurred between groups. Cross-clamp time, VF, ablation procedures, and myocardial hypothermia level are factors that change Tn I and CK-MB releases. In our study, these factors affecting myocardial viability were similar for both groups. However, various studies reported that both types of cardioplegia had equivalent efficacy as a method of myocardial protection during cardiac arrest^[5,15]^.

There was no significant difference regarding the immunoexpression of iNOS, VEGF, and annexin in the myocardium between the Custodiol and blood cardioplegia groups. Custodiol can prevent the inflammatory response of tissue via iNOS molecules because of its myocardial protective effect^[16]^. Immunocytochemical analysis revealed that the cardioprotective and vasodilator molecule, eNOS, was preserved in the atrial tissue in Custodiol and blood cardioplegia groups, but, importantly, it was increased in the Custodiol group. Furthermore, the expression of the Bcl-2 protein in the prevention of apoptosis was also demonstrated in isolated rat myocytes^[17]^. In this study, anti-apoptotic Bcl-2 molecules were well preserved in the myocardial tissue in Custodiol and blood cardioplegia groups, but they were increased in the Custodiol group. Custodiol may contribute to the anti-apoptotic response of tissue via Bcl-2 molecules because of the myocardial protective effect.

This study has potential limitations. First, immunohistochemical studies with myocardial biopsy have high costs; therefore, the sample size is relatively small. There is a lack of evidence in the literature on the subject of our study, so this is a preliminary study. Previously performed animal studies also have small sample sizes. Similar experiments with larger samples should be conducted to obtain more precise results.

## CONCLUSION

Clinical outcomes and cardiac enzyme analysis demonstrated that a single dose of antegrade cold Custodiol cardioplegia and repetitive antegrade blood cardioplegia in elective cardiac surgery were equally effective in protecting the myocardium. However, immunohistochemical studies documented superior cellular oxidative stress resistance and cellular viability with Custodiol infusion. In our opinion, myocardial immunohistochemical analysis needs to be performed with larger series of patients because cardiac histochemical changes can be critical for long-term cardiac performance.

## References

[1] Hölscher M, Groenewoud AF (1991). Current status of the HTK solution of bretschneider in organ
preservation. Transplant Proc.

[2] Bretschneider HJ, Hübner G, Knoll D, Lohr B, Nordbeck H, Spieckermann PG (1975). Myocardial resistance and tolerance to ischemia: physiological
and biochemical basis. J Cardiovasc Surg (Torino).

[3] Bretschneider HJ (1980). Myocardial protection. Thorac Cardiovasc Surg.

[4] Barner HB (1991). Blood cardioplegia: a review and comparison with crystalloid
cardioplegia. Ann Thorac Surg.

[5] Braathen B, Jeppsson A, Scherstén H, Hagen OM, Vengen Ø, Rexius H (2011). One single dose of histidine-tryptophan-ketoglutarate solution
gives equally good myocardial protection in elective mitral valve surgery as
repetitive cold blood cardioplegia: a prospective randomized
study. J Thorac Cardiovasc Surg.

[6] Gatti G, Rauber E, Forti G, Benussi B, Gabrielli M, Gripari C (2019). Safe cross-clamp time using
Custodiol®-histidine-tryptophan-ketoglutarate cardioplegia in the
adult. Perfusion.

[7] Lechner M, Lirk P, Rieder J (2005). Inducible nitric oxide synthase (iNOS) in tumor biology: the two
sides of the same coin. Semin Cancer Biol.

[8] Perkins KA, Pershad S, Chen Q, McGraw S, Adams JS, Zambrano C (2012). The effects of modulating eNOS activity and coupling in
ischemia/reperfusion (I/R). Naunyn Schmiedebergs Arch Pharmacol.

[9] Zhao Y, Vanhoutte PM, Leung SW (2015). Vascular nitric oxide: beyond eNOS. J Pharmacol Sci.

[10] Kanellis J, Paizis K, Cox AJ, Stacker SA, Gilbert RE, Cooper ME (2002). Renal ischemia-reperfusion increases endothelial VEGFR-2 without
increasing VEGF or VEGFR-1 expression. Kidney Int.

[11] Maulik N, Engelman RM, Rousou JA, Flack JE 3rd, Deaton D, Das DK (1999). Ischemic preconditioning reduces apoptosis by upregulating
anti-death gene Bcl-2. Circulation.

[12] de Jong RCM, Pluijmert NJ, de Vries MR, Pettersson K, Atsma DE, Jukema JW (2018). Annexin A5 reduces infarct size and improves cardiac function
after myocardial ischemia-reperfusion injury by suppression of the cardiac
inflammatory response. Sci Rep.

[13] Hachida M, Nonoyama M, Bonkohara Y, Hanayama N, Saitou S, Maeda T (1997). Clinical assessment of prolonged myocardial preservation for
patients with a severely dilated heart. Ann Thorac Surg.

[14] von Oppell UO, Pfeiffer S, Preiss P, Dunne T, Zilla P, Reichart B (1990). Endothelial cell toxicity of solid-organ preservation
solutions. Ann Thorac Surg.

[15] Viana FF, Shi WY, Hayward PA, Larobina ME, Liskaser F, Matalanis G (2013). Custodiol versus blood cardioplegia in complex cardiac
operations: an Australian experience. Eur J Cardiothorac Surg.

[16] Dulak J, Józkowicz A, Dembinska-Kiec A, Guevara I, Zdzienicka A, Zmudzinska-Grochot D (2000). Nitric oxide induces the synthesis of vascular endothelial growth
factor by rat vascular smooth muscle cells. Arterioscler Thromb Vasc Biol.

[17] Misao J, Hayakawa Y, Ohno M, Kato S, Fujiwara T, Fujiwara H (1996). Expression of bcl-2 protein, an inhibitor of apoptosis, and Bax,
an accelerator of apoptosis, in ventricular myocytes of human hearts with
myocardial infarction. Circulation.

